# (^S^ *S*,2*S*,3*R*)-2-(2-Methyl­propane-2-sulfin­amido)-3-phenyl­butyronitrile

**DOI:** 10.1107/S1600536809041233

**Published:** 2009-10-17

**Authors:** Klaus Harms, Michael Marsch, Markus Oberthür, Peter Schüler

**Affiliations:** a Philipps-Universität Marburg, Fachbereich Chemie, Hans-Meerwein-Strasse, D-35032 Marburg, Germany

## Abstract

The absolute configuration has been determined for the title compound, C_14_H_20_N_2_OS. Inter­molecular N—H⋯O hydrogen bonds are observed in the crystal packing, forming infinitive one-dimensional chains with the base vector [100].

## Related literature

For uses of *tert*-butane­sulfinimines, see: Ferreira *et al.* (2009 ▶). For asymmetric Strecker reactions utilizing this auxiliary, see: Davis *et al.* (1994 ▶); Li *et al.* (2003 ▶). For the mannopeptimycin gene cluster, see: Magarvey *et al.* (2006 ▶). For a related structure, see: Harms *et al.* (2009 ▶). 
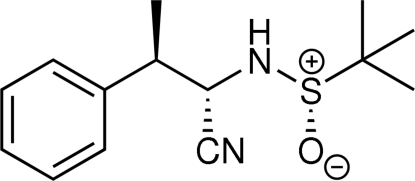


## Experimental

### 

#### Crystal data

C_14_H_20_N_2_OS
*M* *_r_* = 264.38
Orthorhombic, 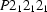

*a* = 8.7892 (3) Å
*b* = 8.7967 (4) Å
*c* = 18.5217 (7) Å
*V* = 1432.02 (10) Å^3^
*Z* = 4
Mo *K*α radiation
μ = 0.22 mm^−1^
*T* = 100 K
0.36 × 0.18 × 0.15 mm

#### Data collection

STOE IPDS II diffractometer
Absorption correction: none
22029 measured reflections
3031 independent reflections
2624 reflections with *I* > 2σ(*I*)
*R*_int_ = 0.070

#### Refinement

*R*[*F* ^2^ > 2σ(*F* ^2^)] = 0.029
*wR*(*F* ^2^) = 0.064
*S* = 0.92
3031 reflections
244 parameters
All H-atom parameters refined
Δρ_max_ = 0.18 e Å^−3^
Δρ_min_ = −0.23 e Å^−3^
Absolute structure: Flack (1983 ▶), 1272 Friedel pairs
Flack parameter: 0.02 (6)

### 

Data collection: *X-AREA* (Stoe & Cie, 2002 ▶); cell refinement: *X-AREA*; data reduction: *X-AREA*; program(s) used to solve structure: *SIR2004* (Burla *et al*, 2005 ▶); program(s) used to refine structure: *SHELXL97* (Sheldrick, 2008 ▶); molecular graphics: *DIAMOND* (Brandenburg, 1999 ▶); software used to prepare material for publication: *publCIF* (Westrip, 2009 ▶).

## Supplementary Material

Crystal structure: contains datablocks global, I. DOI: 10.1107/S1600536809041233/pv2212sup1.cif

Structure factors: contains datablocks I. DOI: 10.1107/S1600536809041233/pv2212Isup2.hkl

Additional supplementary materials:  crystallographic information; 3D view; checkCIF report

## Figures and Tables

**Table 1:** Hydrogen-bond geometry (Å, °)

*D*—H⋯*A*	*D*—H	H⋯*A*	*D*⋯*A*	*D*—H⋯*A*
N1—H01⋯O1^i^	0.89 (2)	2.167 (19)	2.9511 (18)	146.5 (18)

Symmetry code: (i) 
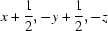
.

**Fig. 1.**
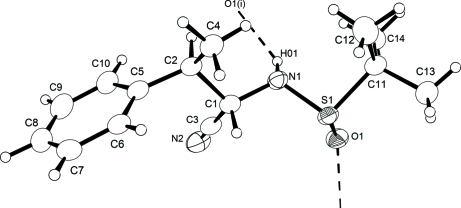
A view of (I). Displacement ellipsoids are drawn at the 50% probability level. Symmetry operation (i): x+1/2, -y+1/2, -z.

**Fig. 2.**
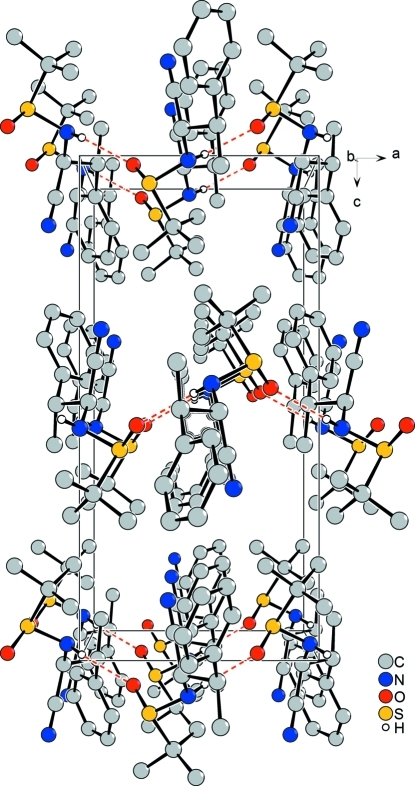
Unit cell packing of (I) viewed down the b-axis. Dotted lines indicate hydrogen bonds.

## supplementary crystallographic information

### Comment

Chiral sulfinimines have proven to be powerful and versatile precursors for the synthesis of nonproteinogenic amino acids (Ferreira *et al.*, 2008). They allow the stereoselective introduction of cyanide therefore representing an asymmetric modification of the Strecker reaction (Davis *et al.*, 1994); Li *et al.*, 2003). We have synthesized the title compound, (I), that can be hydrolyzed to give (2*S*,3*R*)-β-methylphenylalanine which is of practical use as reference substance in the investigation of the methyltransferase present in the mannopeptimycin gene cluster (Magarvey *et al.*, 2006). In this paper we report the crystal structure and absolute configuration of (I).

The molecular structure of (I) is presented in Fig. 1. The structure exhibits intermolecular N—H···O hydrogen bonds [H···O = 2.167 (19) Å] resulting in infinitive one dimensional chains with the base vector [1 0 0] (details have have been provided in Table 1 and Fig. 2).

The crystal structure and absolute configuration of a closely related compound has just been reported (Harms *et al.*, 2009).

### Experimental

Trimethylsilyl cyanide (TMSCN) (706 µ*L*, 5.64 mmol) was added dropwise to a solution of (*^S^S*)-(2-phenylpropyliden)-2-methyl-2-propansulfinylimin (1.12 g, 4.70 mmol) and CsF (858 mg, 5.64 mmol) in 50 ml *n*-hexane at 240 K. The mixture was stirred at this temperature for 14 h and subsequently quenched with semisaturated aqueous NH_4_Cl solution. Extraction with EtOAc (2×50 ml) and drying of the combined organic phases (MgSO_4_) yielded a crude mixture of 3*S*/3*R* epimers. Crystallization from petrolether/EtOAc yielded 370 mg (1.41 mmol, 35%) of a 1:1 mixture of the diastereomers. Flash column chromatography of the mother liquor yielded 80 mg (303 mmol, 6%) of the pure 3*S *isomer, which had a slightly higher *R*_f_-value (*R*_f_= 0.30 in petrol ether/EtOAc 2:1) than the 3*R* isomer of which 60 mg (227 mmol, 5%) could be isolated. The remaining fractions afforded 400 mg (1.53 mmol, 32%) of a roughly 1:1 mixture of the epimers. (*^S^S*,2*S*,3*R*)-(2-Methylpropansulfinyl)-2-amino-3-phenylbutyronitril was crystallized from petrol ether/THF.

### Refinement

H atoms were located in the difference Fourier map and all H atom parameters were allowed to refine with isotropic displacement parameters.

### Crystal data

**Table:**

C_14_H_20_N_2_OS	*F*(000) = 568
*M**_r_* = 264.38	*D*_x_ = 1.226 Mg m^−^^3^
Orthorhombic, *P*2_1_2_1_2_1_	Mo *K*α radiation, λ = 0.71073 Å
Hall symbol: P 2ac 2ab	Cell parameters from 22507 reflections
*a* = 8.7892 (3) Å	θ = 2.2–25°
*b* = 8.7967 (4) Å	µ = 0.22 mm^−^^1^
*c* = 18.5217 (7) Å	*T* = 100 K
*V* = 1432.02 (10) Å^3^	Prism, colourless
*Z* = 4	0.36 × 0.18 × 0.15 mm

### Data collection

**Table:**

STOE IPDS II diffractometer	2624 reflections with *I* > 2σ(*I*)
Radiation source: sealed X-ray tube	*R*_int_ = 0.070
graphite	θ_max_ = 26.8°, θ_min_ = 2.2°
area detetor, ω scans	*h* = −11→11
22029 measured reflections	*k* = −11→11
3031 independent reflections	*l* = −23→23

### Refinement

**Table:**

Refinement on *F*^2^	Hydrogen site location: difference Fourier map
Least-squares matrix: full	All H-atom parameters refined
*R*[*F*^2^ > 2σ(*F*^2^)] = 0.029	*w* = 1/[σ^2^(*F*_o_^2^) + (0.0378*P*)^2^] where *P* = (*F*_o_^2^ + 2*F*_c_^2^)/3
*wR*(*F*^2^) = 0.064	(Δ/σ)_max_ < 0.001
*S* = 0.92	Δρ_max_ = 0.18 e Å^−^^3^
3031 reflections	Δρ_min_ = −0.23 e Å^−^^3^
244 parameters	Extinction correction: *SHELXL97* (Sheldrick, 2008)
0 restraints	Extinction coefficient: 0.0113 (13)
0 constraints	Absolute structure: Flack (1983), 1272 Friedel pairs
Secondary atom site location: difference Fourier map	Flack parameter: 0.02 (6)

### Special details

**Table:**

Experimental. δ_H_(300 MHz; DMSO) 1.13 (s, 9H, *t*Bu), 1.28 (d, 3H, ^3^*J*_Me,CH _= 7.0 Hz, CH_3_), 3.14 (dq, 1H, ^3^*J*_CH,CHN _= 9.9, *J*_CH,Me _= 7.0 Hz, CH), 4.48 (pt, 1H, ^3^*J*_CHN,CH _= 9.9 Hz, CHN), 6.37 (d, 1H, ^3^*J*_NH,CHN _= 9.9 Hz, NH), 7.22 – 7.38 (m, 5H, CH_arom_); δ_C_(75 MHz; DMSO-d_6_) 18.3 (CH_3_), 22.5 (C(CH_3_)_3_), 43.6 (CH), 52.4 (CHN), 56.4 (C(CH_3_)_3_), 119.8 (CN), 127.2 (*p*-CH_arom_), 127.8 (CH_arom_),128.5 (CH_arom_), 141.7 (*i*-C_arom_).
Geometry. All s.u.'s (except the s.u. in the dihedral angle between two l.s. planes) are estimated using the full covariance matrix. The cell s.u.'s are taken into account individually in the estimation of s.u.'s in distances, angles and torsion angles; correlations between s.u.'s in cell parameters are only used when they are defined by crystal symmetry. An approximate (isotropic) treatment of cell s.u.'s is used for estimating s.u.'s involving l.s. planes.
Refinement. Refinement of *F*^2^ against ALL reflections. The weighted *R*-factor *wR* and goodness of fit *S* are based on *F*^2^, conventional *R*-factors *R* are based on *F*, with *F* set to zero for negative *F*^2^. The threshold expression of *F*^2^ > 2σ(*F*^2^) is used only for calculating *R*-factors(gt) *etc*. and is not relevant to the choice of reflections for refinement. *R*-factors based on *F*^2^ are statistically about twice as large as those based on *F*, and *R*- factors based on ALL data will be even larger.

### Fractional atomic coordinates and isotropic or equivalent isotropic displacement parameters (Å^2^)

**Table:**

	*x*	*y*	*z*	*U*_iso_*/*U*_eq_	
C1	0.43208 (18)	0.5330 (2)	−0.00956 (9)	0.0303 (4)	
C2	0.56755 (17)	0.6458 (2)	−0.00597 (9)	0.0311 (4)	
C3	0.40206 (16)	0.4817 (2)	−0.08405 (11)	0.0337 (4)	
C4	0.57924 (19)	0.7071 (2)	0.07072 (10)	0.0343 (4)	
C5	0.55157 (17)	0.7666 (2)	−0.06356 (9)	0.0322 (4)	
C6	0.46159 (19)	0.8950 (2)	−0.05357 (10)	0.0362 (4)	
C7	0.4397 (2)	0.9986 (2)	−0.10900 (12)	0.0451 (5)	
C8	0.5075 (2)	0.9750 (3)	−0.17589 (12)	0.0480 (5)	
C9	0.5996 (2)	0.8499 (3)	−0.18570 (11)	0.0467 (5)	
C10	0.6223 (2)	0.7472 (2)	−0.13039 (10)	0.0394 (4)	
C11	0.39529 (17)	0.2377 (2)	0.15401 (9)	0.0321 (4)	
C12	0.4864 (3)	0.3546 (3)	0.19584 (12)	0.0498 (5)	
C13	0.2643 (2)	0.1791 (3)	0.20040 (12)	0.0447 (5)	
C14	0.4918 (2)	0.1082 (3)	0.12683 (12)	0.0459 (5)	
N1	0.45589 (15)	0.40435 (17)	0.03876 (8)	0.0302 (3)	
N2	0.37703 (17)	0.4385 (2)	−0.14134 (9)	0.0449 (4)	
O1	0.23286 (12)	0.20929 (14)	0.03335 (7)	0.0376 (3)	
S1	0.30144 (4)	0.33362 (5)	0.07741 (2)	0.03041 (11)	
H2	0.6626 (19)	0.578 (2)	−0.0175 (9)	0.034 (5)*	
H4A	0.655 (2)	0.795 (2)	0.0753 (11)	0.042 (5)*	
H8	0.495 (2)	1.053 (3)	−0.2164 (11)	0.059 (6)*	
H01	0.521 (2)	0.334 (2)	0.0226 (10)	0.038 (5)*	
H4B	0.486 (2)	0.751 (2)	0.0859 (10)	0.041 (5)*	
H10	0.689 (2)	0.649 (2)	−0.1366 (10)	0.049 (5)*	
H1	0.3383 (18)	0.586 (2)	0.0075 (9)	0.030 (4)*	
H13A	0.307 (3)	0.113 (3)	0.2399 (12)	0.064 (7)*	
H6	0.417 (2)	0.910 (2)	−0.0079 (10)	0.040 (5)*	
H14C	0.570 (2)	0.146 (2)	0.0968 (11)	0.053 (6)*	
H12A	0.509 (3)	0.315 (3)	0.2468 (13)	0.063 (6)*	
H14B	0.425 (2)	0.024 (3)	0.0969 (13)	0.059 (7)*	
H4C	0.604 (2)	0.621 (2)	0.1034 (11)	0.051 (6)*	
H13B	0.205 (3)	0.270 (3)	0.2212 (12)	0.067 (7)*	
H12C	0.576 (3)	0.393 (3)	0.1711 (14)	0.080 (8)*	
H7	0.379 (2)	1.088 (3)	−0.0992 (12)	0.053 (6)*	
H12B	0.424 (3)	0.447 (3)	0.2040 (12)	0.060 (7)*	
H9	0.650 (2)	0.827 (3)	−0.2315 (12)	0.060 (6)*	
H14A	0.539 (3)	0.049 (3)	0.1672 (12)	0.061 (7)*	
H13C	0.192 (3)	0.115 (3)	0.1726 (12)	0.061 (6)*	

### Atomic displacement parameters (Å^2^)

**Table:**

	*U*^11^	*U*^22^	*U*^33^	*U*^12^	*U*^13^	*U*^23^
C1	0.0237 (8)	0.0324 (9)	0.0348 (9)	0.0004 (7)	−0.0002 (7)	−0.0003 (7)
C2	0.0230 (7)	0.0334 (10)	0.0368 (9)	−0.0003 (7)	0.0000 (6)	0.0008 (8)
C3	0.0232 (7)	0.0387 (9)	0.0394 (10)	−0.0018 (6)	0.0012 (7)	−0.0008 (9)
C4	0.0303 (8)	0.0346 (10)	0.0381 (10)	−0.0040 (7)	−0.0005 (7)	−0.0012 (8)
C5	0.0242 (7)	0.0346 (9)	0.0378 (9)	−0.0056 (7)	−0.0021 (6)	−0.0003 (8)
C6	0.0240 (8)	0.0381 (10)	0.0465 (10)	−0.0054 (7)	−0.0032 (7)	0.0046 (8)
C7	0.0304 (9)	0.0389 (11)	0.0661 (14)	−0.0059 (8)	−0.0134 (9)	0.0087 (10)
C8	0.0445 (10)	0.0477 (13)	0.0518 (12)	−0.0203 (9)	−0.0177 (9)	0.0157 (10)
C9	0.0468 (10)	0.0524 (13)	0.0408 (11)	−0.0187 (10)	−0.0043 (8)	0.0025 (11)
C10	0.0368 (9)	0.0407 (11)	0.0408 (10)	−0.0094 (8)	0.0004 (7)	0.0008 (9)
C11	0.0264 (8)	0.0318 (9)	0.0380 (9)	−0.0014 (7)	0.0002 (6)	−0.0011 (8)
C12	0.0603 (12)	0.0454 (13)	0.0436 (11)	−0.0135 (11)	−0.0102 (10)	0.0023 (11)
C13	0.0351 (9)	0.0486 (12)	0.0505 (11)	0.0009 (10)	0.0047 (8)	0.0134 (11)
C14	0.0428 (10)	0.0481 (12)	0.0466 (11)	0.0145 (9)	0.0039 (9)	0.0072 (10)
N1	0.0235 (6)	0.0296 (8)	0.0375 (8)	0.0017 (6)	0.0030 (6)	0.0012 (7)
N2	0.0350 (8)	0.0575 (11)	0.0421 (10)	−0.0049 (8)	0.0003 (7)	−0.0064 (8)
O1	0.0311 (6)	0.0367 (7)	0.0451 (7)	−0.0088 (5)	−0.0069 (5)	−0.0010 (6)
S1	0.02191 (15)	0.0315 (2)	0.0378 (2)	−0.00129 (16)	0.00057 (16)	0.0009 (2)

### Geometric parameters (Å, °)

**Table:**

C1—N1	1.458 (2)	C9—C10	1.380 (3)
C1—C3	1.476 (3)	C9—H9	0.98 (2)
C1—C2	1.551 (2)	C10—H10	1.05 (2)
C1—H1	0.998 (17)	C11—C14	1.507 (3)
C2—C5	1.512 (2)	C11—C12	1.517 (3)
C2—C4	1.523 (2)	C11—C13	1.526 (2)
C2—H2	1.046 (18)	C11—S1	1.8454 (17)
C3—N2	1.148 (2)	C12—H12A	1.02 (2)
C4—H4A	1.019 (19)	C12—H12C	0.97 (3)
C4—H4B	0.95 (2)	C12—H12B	0.99 (3)
C4—H4C	0.99 (2)	C13—H13A	1.01 (2)
C5—C6	1.391 (3)	C13—H13B	1.03 (2)
C5—C10	1.395 (2)	C13—H13C	0.99 (2)
C6—C7	1.386 (3)	C14—H14C	0.94 (2)
C6—H6	0.943 (19)	C14—H14B	1.10 (2)
C7—C8	1.390 (3)	C14—H14A	1.00 (2)
C7—H7	0.96 (2)	N1—S1	1.6560 (14)
C8—C9	1.378 (3)	N1—H01	0.89 (2)
C8—H8	1.02 (2)	O1—S1	1.4918 (12)
			
N1—C1—C3	111.21 (15)	C9—C10—C5	120.9 (2)
N1—C1—C2	111.10 (13)	C9—C10—H10	122.5 (11)
C3—C1—C2	111.90 (13)	C5—C10—H10	116.5 (11)
N1—C1—H1	106.6 (10)	C14—C11—C12	112.71 (17)
C3—C1—H1	106.9 (9)	C14—C11—C13	110.95 (17)
C2—C1—H1	108.8 (10)	C12—C11—C13	109.88 (17)
C5—C2—C4	114.53 (15)	C14—C11—S1	109.91 (13)
C5—C2—C1	110.36 (13)	C12—C11—S1	108.57 (14)
C4—C2—C1	108.55 (13)	C13—C11—S1	104.48 (11)
C5—C2—H2	109.2 (10)	C11—C12—H12A	110.1 (15)
C4—C2—H2	109.7 (9)	C11—C12—H12C	115.1 (16)
C1—C2—H2	104.0 (10)	H12A—C12—H12C	113 (2)
N2—C3—C1	178.3 (2)	C11—C12—H12B	109.8 (13)
C2—C4—H4A	112.9 (11)	H12A—C12—H12B	104 (2)
C2—C4—H4B	111.2 (11)	H12C—C12—H12B	104 (2)
H4A—C4—H4B	103.3 (15)	C11—C13—H13A	108.8 (13)
C2—C4—H4C	108.2 (12)	C11—C13—H13B	109.2 (12)
H4A—C4—H4C	112.6 (15)	H13A—C13—H13B	111.5 (17)
H4B—C4—H4C	108.6 (16)	C11—C13—H13C	112.4 (13)
C6—C5—C10	118.05 (17)	H13A—C13—H13C	106.7 (17)
C6—C5—C2	121.98 (15)	H13B—C13—H13C	108.3 (18)
C10—C5—C2	119.90 (16)	C11—C14—H14C	109.8 (13)
C7—C6—C5	120.94 (19)	C11—C14—H14B	112.1 (11)
C7—C6—H6	120.9 (12)	H14C—C14—H14B	109.4 (17)
C5—C6—H6	118.2 (12)	C11—C14—H14A	112.4 (13)
C6—C7—C8	120.2 (2)	H14C—C14—H14A	108.6 (17)
C6—C7—H7	118.1 (14)	H14B—C14—H14A	104.4 (18)
C8—C7—H7	121.7 (14)	C1—N1—S1	116.06 (11)
C9—C8—C7	119.2 (2)	C1—N1—H01	115.0 (12)
C9—C8—H8	120.2 (12)	S1—N1—H01	114.2 (13)
C7—C8—H8	120.4 (12)	O1—S1—N1	111.73 (7)
C8—C9—C10	120.6 (2)	O1—S1—C11	105.41 (7)
C8—C9—H9	122.8 (14)	N1—S1—C11	97.92 (7)
C10—C9—H9	116.6 (14)		
			
N1—C1—C2—C5	172.20 (13)	C7—C8—C9—C10	1.3 (3)
C3—C1—C2—C5	47.23 (18)	C8—C9—C10—C5	0.6 (3)
N1—C1—C2—C4	−61.53 (18)	C6—C5—C10—C9	−2.1 (3)
C3—C1—C2—C4	173.50 (14)	C2—C5—C10—C9	174.84 (15)
N1—C1—C3—N2	31 (6)	C3—C1—N1—S1	−88.88 (15)
C2—C1—C3—N2	156 (6)	C2—C1—N1—S1	145.76 (12)
C4—C2—C5—C6	−39.8 (2)	C1—N1—S1—O1	90.88 (13)
C1—C2—C5—C6	83.09 (19)	C1—N1—S1—C11	−158.98 (12)
C4—C2—C5—C10	143.46 (15)	C14—C11—S1—O1	48.56 (14)
C1—C2—C5—C10	−93.69 (17)	C12—C11—S1—O1	172.26 (13)
C10—C5—C6—C7	1.6 (2)	C13—C11—S1—O1	−70.54 (14)
C2—C5—C6—C7	−175.21 (15)	C14—C11—S1—N1	−66.65 (14)
C5—C6—C7—C8	0.2 (3)	C12—C11—S1—N1	57.04 (15)
C6—C7—C8—C9	−1.7 (3)	C13—C11—S1—N1	174.25 (13)

### Hydrogen-bond geometry (Å, °)

**Table:**

*D*—H···*A*	*D*—H	H···*A*	*D*···*A*	*D*—H···*A*
N1—H01···O1^i^	0.89 (2)	2.167 (19)	2.9511 (18)	146.5 (18)

Symmetry codes: (i) *x*+1/2, −*y*+1/2, −*z*.
